# A deep investigation of the poorly studied open cluster King 18 using CCD VRI, Gaia DR3 and 2MASS

**DOI:** 10.1038/s41598-024-70133-y

**Published:** 2024-10-10

**Authors:** Nasser M. Ahmed, R. Bendary, R. M. Samir, E. G. Elhosseiny

**Affiliations:** National Research Institute of Astronomy and Geophysics (NRIAG), Helwan, Cairo 11421 Egypt

**Keywords:** Star cluster, Gaia DR3, 2Mass, CMD, Parallax, Proper motion, Distance, Membership, Astronomy and astrophysics, Micro-optics, X-rays

## Abstract

In this paper, we re-estimate the astrometric and photometric parameters of the young open star cluster King 18 based on Gaia Data Release 3 (DR3), Two Micron All-sky Survey (2MASS) and VRI CCD observations using the f/4.9 Newtonian focus of 74-inch telescope at Kottamia Astronomical Observatory (KAO) in Egypt. King 18 is a poorly studied open star cluster, for which new results are found in the current study. In order to estimate the membership and determine all the astrophysical parameters of the cluster, we have used data from Gaia DR3 and KAO. The center, cluster radius, radial density distribution, color-magnitude diagrams, distance, age, and reddening of King 18 are calculated. Also, the luminosity and mass functions, the total mass and the relaxation time of the cluster are estimated. The slope value of the mass function ($\alpha$) of King 18 is found to be 2.27± 0.17, which is comparable with Salpeter value. Our estimates for the average cluster age and the relaxation time are 224 ± 6.3 and 28.92 Myrs, respectively. This indicates that King 18 is dynamically stable and a relaxed cluster. The cluster distance modulus from Gaia, 2Mass and VRI observations has been determined to be 12.380 ± 1.320, 12.320 ± 0.107 and 12.280 ± 0.290 mag respectively, which corresponds to distances of 2992.26, 2910.72 and 2857.59 pc, respectively. These results are in good agreement within the error. Moreover the color excesses E(V–I), E(J–$\hbox {K}_s$) and E($\hbox {G}_{{BP}}$–$\hbox {G}_{{RP}}$) are 0.850 ± 0.087, 0.380 ± 0.091 and 0.980 ± 0.130 respectively. Finally, the proper motions ($\mu _{\alpha }$cos$\delta$, $\mu _{\delta }$), and parallaxes ($\varpi$) are $-2.603 \pm 0.018$, $-2.106 \pm 0.013$ and 0.324 ± 0.040, respectively.

## Introduction

Star clusters are considered key objects for our understanding of stellar evolution and galactic structure. To resolve the formation history of the Milky Way disc, it is important to study open star clusters, groups of stars with the same age and abundance pattern that are grasped together by mutual gravitation. Open Clusters (OCs) are homogeneous stellar systems each of whose component stars formed at essentially the same time and under the same physical conditions, making them good tracers of changing conditions in interstellar medium. They contain from a few dozens to a few thousands stars located at comparable distances. OCs are beneficial objects to understand the structure, kinematics and features of the Milky Way^1–4^. Every cluster contains stars with different masses that were originated from the collapse of the same dense molecular cloud and thus share the same age, kinematics and chemical composition.

OCs were the topic of many studies in recent years. They are frequently used to recognize the Galactic disk properties, such as studying the spiral arms of the Milky Way^5–7^, stellar structure and star formation process^8–10^, chemical homogeneity and age-metallicity relation^11–16^.

OCs are spread throughout the Milky Way disk and they show vast ranges in ages (from < 100 Myr to $\sim$ 8 Gyr)^3,17,18^. The distributions of the physical parameters of OCs such as mass, age, and size are governing their formation and evolution (for a recent review, see^10^). The main astrophysical parameters of OCs such as metallicity, color excess, age, extinction, and distance can be obtained from the color-magnitude diagrams (CMDs) by comparing with stellar models, such as isochrones.

The accurate estimation of cluster member stars beside using homogeneous data and procedures during analysis, is important to determine rigorously the astrophysical parameters of the cluster. Different authors studied the same open cluster and they found quite different values for astrophysical parameters^17,19,20^. The determination of cluster parameters is affected by the consolidation of data with varying levels of quality then applied to isochrones fitting methods, cluster membership determination, and analysis methods^21,22^.

King 18 is located towards the Perseus spiral arm at $\alpha$ = ${22}^h$ ${52.3}^m$ and $\delta$ = ${58}^\circ$ $18'$ (J2000.0), which corresponds to Galactic coordinates of l = 107.8$^\circ$ and b = ${1.0}^\circ$. It was discovered by^23^ and was described as poor stellar cluster with diameter of 4 arcmin. Based on^17^, the angular diameter of King 18 is found to be $5 '$. To our knowledge, the first study of this cluster was carried out by^24^, who calculated some cluster parameters based on BV photometry and near IR data from 2MASS. He found that King 18 is located at a distance of 2.34 kpc and calculated the following; reddening E(B - V) = 0.63 mag, age = 251 Myr, the total cluster mass = 1050 $\hbox {M}_\odot$ and the angular diameter was found to be three times greater than the value recorded in literature. Also, some structural parameters of King 18 were determined by^25^, where the cluster age was found to be 350 Myr, the cluster distance was calculated as 1860 ± 85 pc, and color excess E(B-V) was found to be 0.52 mag. On the other side, Glushkova et al.^26^ presented different values of structural parameters of King 18 in comparison with^25^. They calculated its age as 130 ± 10 Myr, its distance as 3010 pc, and its color excess as 0.69 ± 0.04 mag.

It is obvious that there is a difference in the parameters of King 18 from a study to another. So there is a requirement to make a journey back to this cluster and estimate its parameters in a more precise way by using new data and tools of analysis. In the current study, we use VRI CCD photometric observations of King18 using the f/4.9 1.88 M telescope at Kottamia Astronomical Observatory (KAO) and Gaia DR3 database to estimate the astrometric and astrophysical parameters of King 18.

The paper is organized as follows: data extraction, observations and data reductions are introduced in “Data extraction, observations and data reductions”. The estimation of cluster density profile and cluster radius is presented in “Cluster density profile and radius”. “Proper motion, membership determination and cluster center” describes the study of proper motion, determination of membership of stars in the cluster and estimating the cluster center. In “The color magnitude diagrams and cluster age”, color magnitude diagrams and cluster age are discussed. Luminosity, mass functions and dynamical state of the cluster are illustrated in “Luminosity, the cluster mass, mass functions and dynamical state”. Finally, conclusion of this study is presented in “Summary and conclusions”.

## Data extraction, observations and data reductions

**Figure 1:**
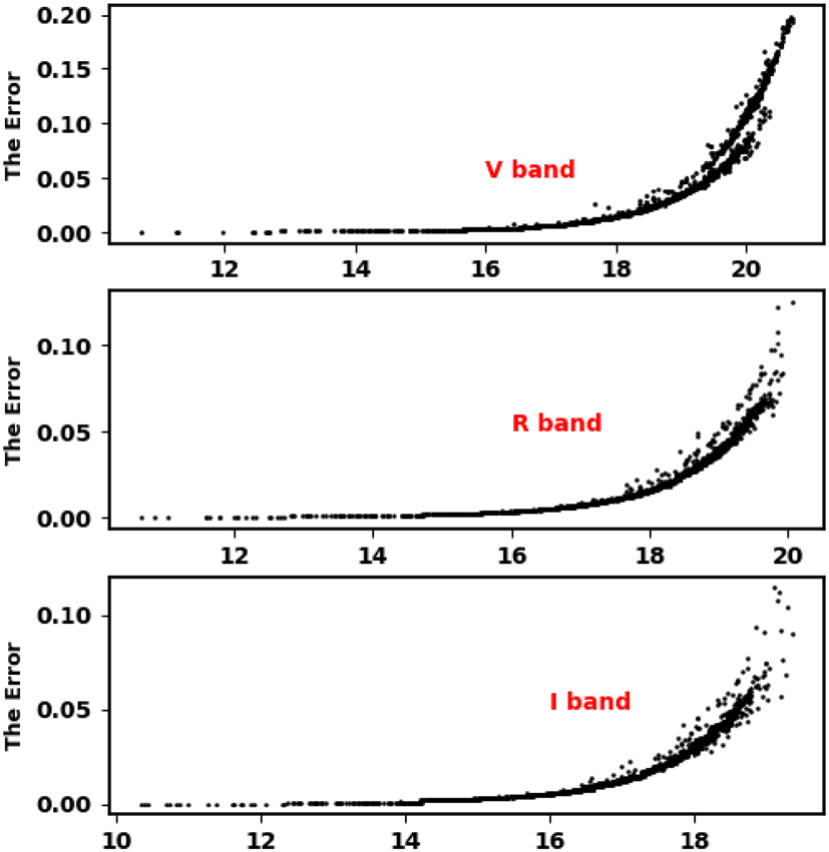
The VRI errors of the observed magnitudes for the stars of King 18.

In our current work, we use VRI CCD photometric observations from KAO, Gaia DR3 database and 2Mass (Fig. 1).

### Gaia DR3 data

**Figure 2:**
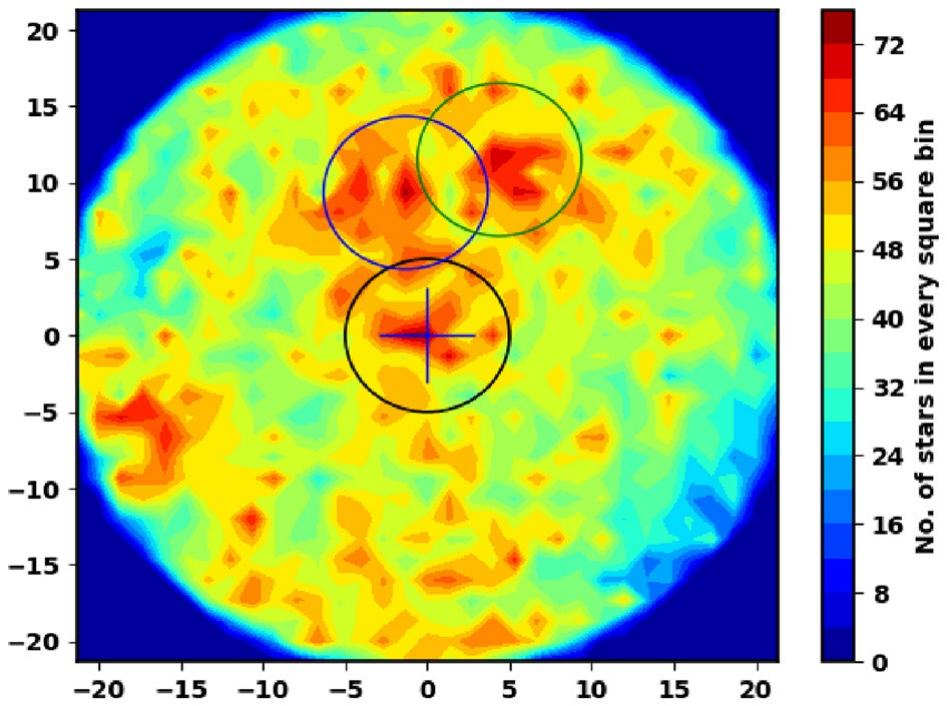
The number surface density of King 18 using the data of Gaia DR3. The cross-hairs represent the center of King 18 cluster. The green and blue circles are stars batches around the cluster. They might be companions to King 18 (the running future work).

**Figure 3:**
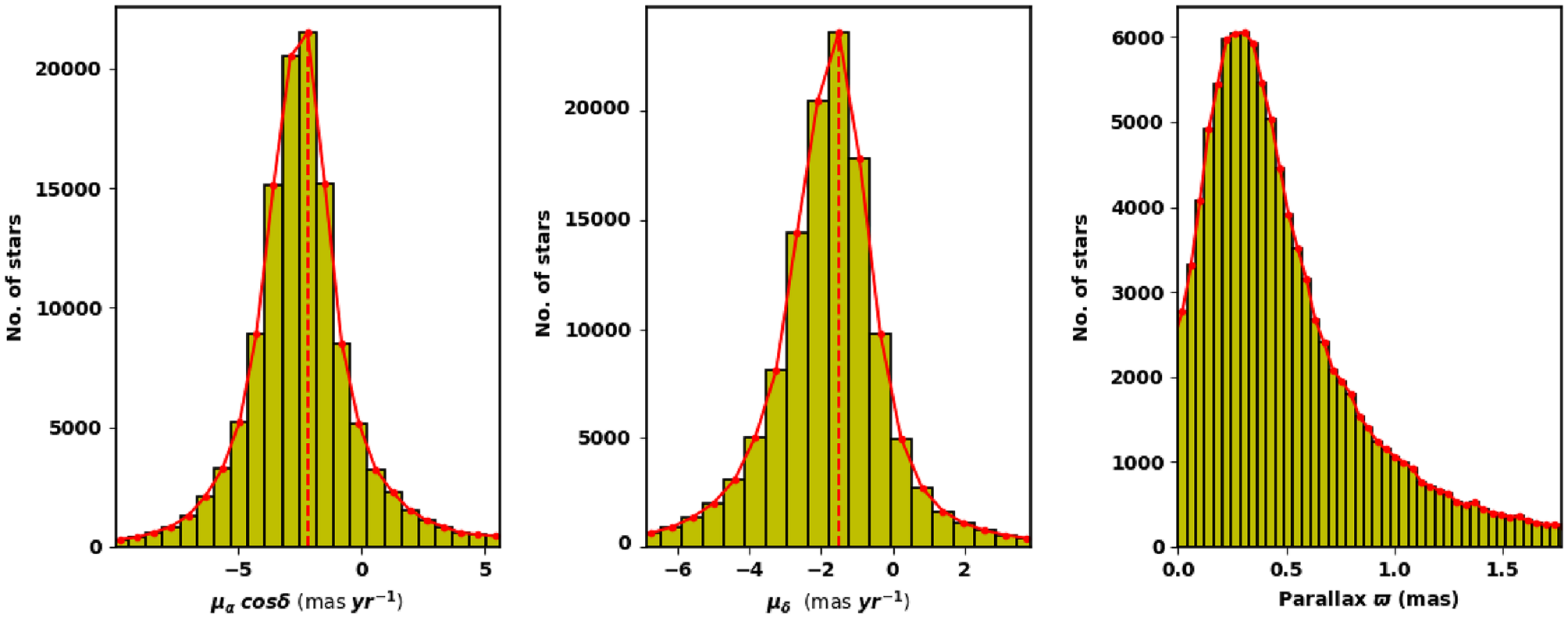
The proper motion in right ascension, declination, and parallax in the field of King 18.

We extract the archived data of King 18 from Gaia **(Gaia DR3)**^27^. This database consists of positions on the sky ($\alpha$, $\delta$), proper motions ($\mu _{\alpha }$cos$\delta$, $\mu _{\delta }$), and parallaxes with a limiting magnitude of G = 21 mag. Gaia DR3 provides astrophysical parameters for many celestial objects derived from parallaxes, broadband photometry, and mean radial velocity spectra. In Gaia DR3, the trigonometric parallax errors are 0.02-0.07 milliarcsecond (mas) for sources at G $\le$ 17 mag, 0.5 mas for G = 20 mag and reach 1.3 mas for G = 21 mag. The proper motion errors are 0.02-0.07 mas $\hbox {year}^{-1}$, reaching up to 0.5 mas $\hbox {year}^{-1}$ for G = 20 mag and 1.4 mas $\hbox {year}^{-1}$ for G = 21 mag. Moreover, it contains G magnitudes for around 1.806 billion sources and $G_{BP}$ and $\hbox {G}_{{RP}}$ magnitudes for around 1.542 billion and 1.555 billion sources, respectively. Fig. 2 shows the number surface density of King 18 from Gaia DR3 and Fig. 3 shows the proper motions $\mu _{\alpha }$cos$\delta$, $\mu _{\delta }$ and parallax $\varpi$ histograms.

### 2MASS data

In this section, we have used the Two Micros All-sky Survey (2MASS^28^) data for the cluster King 18. This data set uses two highly automated 1.3 m telescopes, one at Mt. Hopkins, Arizona (AZ), USA and the other at CTIO, Chile, with a 3-channel camera (256 $\times$ 256 array of HgCdTe detectors in each channel). The 2MASS catalog provides J (1.25 $\upmu$m), H (1.65 $\upmu$m) and $\hbox {K}_s$ (2.17 $\upmu$m) band photometry for millions of galaxies and nearly a half-billion stars. The sensitivity of this catalog is 15.8 mag for J, 15.1 mag for H and 14.3 mag for $\hbox {K}_s$ band at S/N =10.

### VRI photometric observations

The 74-inch telescope of the KAO, was utilized to make the VRI CCD photometric observations of King 18, that are used in this investigation. The observation has been secured in the Newtonian focus with a plate scale of 22.53 arcsec/mm and field area of 10 $\times$ 10 $\hbox {arcmin}^2$ on the night of August 27, 2014. The different characteristics of the CCD Camera used in KAO are explained in details, see^29^.

The numbers of observed stars in V, R, and I bands are 1772, 2576, and 3910, respectively. The mean error for each band is found to be 0.027, 0.024 and 0.023, respectively.

#### Photometric calibration

For calibration purposes, the observational raw data of king 18 was observed by the f/4.9 Newtonian focus of 74-inches KAO telescope. In addation, there are 9 dome flats for every filter and 10 bias frames. The Processing and analysis of the data were done by Python programming language, utilizing several modules and SExtractor function^30^. First of all, we began the processing by using the ccdproc package^31^, which is an Astropy-affiliated package. This process included bias subtraction, flat field correction and cosmic ray removal with the L.A.Cosmic algorithm in the lacosmic Python package^32^. The stars have been detected by using the SExtractor and then Astropy package^33^ is applied. Using the background estimated map for each frame, the Photutils package, which offers tools for photometry of astronomical sources^34^, is applied to obtain instrumental magnitudes. Next, we added World Coordinate System (WCS) information to each frame using the Astrometry.net tool^35^. This is important because it allows us to cross-match the sources with catalog data for additional calibration.

Finally, STDPipe^36^, a suite of Python scripts designed for astrometry, photometry, and transient detection tasks in optical imagery, was employed to compute the calibrated magnitudes for all stars within the observed field. The detected stars were matched with Pan-STARRS1 (PS1) catalogue^37^ and then the photometric model for their instrumental magnitudes was built. This photometric model for the calibrated magnitudes, $mag_{Cal}$, was defined as follows, based on^36^:-

1 $$\begin{aligned} mag_{Cal} = mag_{Inst} + ZP(X,Y) + C \end{aligned}$$

Here, $mag_{Inst}$ is the instrumental magnitude of the source measured by the detector, ZP is the spatially varying zero-point function, and C is a color-correction term to account for the color distribution of the PS1 calibration stars and the resulting errors are shown in Fig. 1. In this work, the magnitudes of PS1 stars are converted to Johnson-Cousins filters (UBVRI) by using equations of^38^.

## Cluster density profile and radius

To study the cluster structure and to construct radial density profile, the first step is to find rigorously the cluster center. Our major goal is to estimate the highest central density of stars in the cluster. Subsequently, we have created the two dimensional-histogram of star counts in both right ascension ($\alpha$) and declination ($\delta$) using the Gaia DR3 database. We used the function histogram2d in numpy package and found the cell with maximum stars. We repeated the process in “Proper motion, membership determination and cluster center”, this time focusing solely on member stars, and discovered no difference.

**Figure 4:**
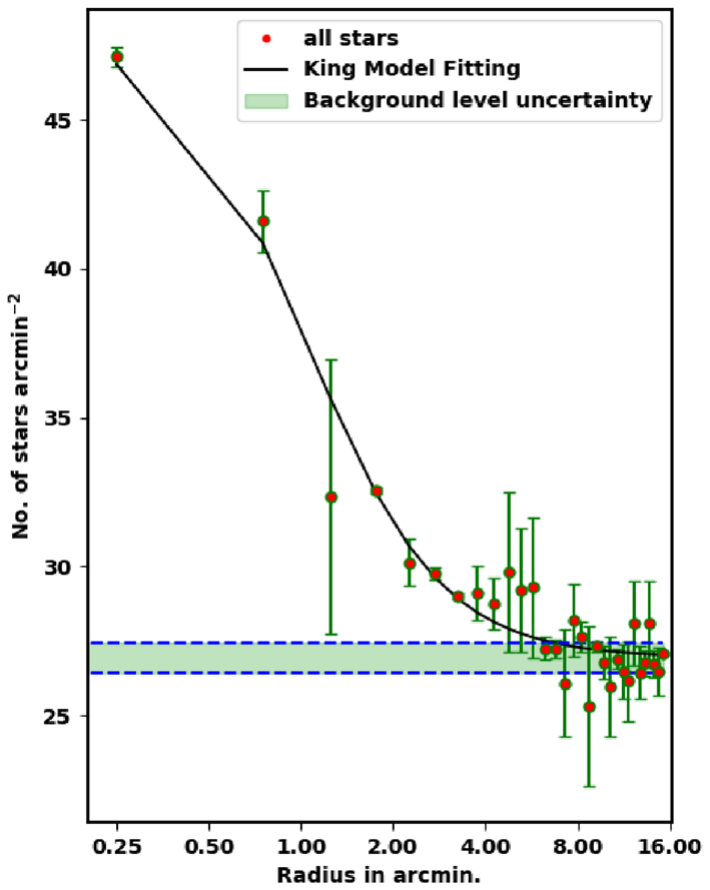
The radial density profile (RDP) of King 18.

To appraise the cluster extent, we construct the radial density profile (RDP) of King 18 through splitting its observed area into concentric circles. The number of stars is counted in every shell or ring as $N_i$, then the star number density is calculated as $f_i = N_i /A_{i}$ where $A_i$ is the ring or shell area ($\pi (R^2_{i+1} -R^2_{i} )$ ) and $R_i$ and $R_{i+1}$ represent the inner and outer radius. The RDP is presented in Fig. 4, where the black solid line shows the fitted King model of^39^. Thus the density function *f(r)* is expressed as :

2 $$\begin{aligned} f(r)= f_{bg} + \dfrac{f_{o}}{1+(r/r_{c})^{2}} \end{aligned}$$

where ${r}_c$ , ${f}_{{bg}}$ , and ${f}_0$ are the core radius, the background density, and the central density of the cluster, respectively. By fitting the King model to the RDP *f*(*r*), we can evaluate the structural parameters for King 18. The blue dashed line in Fig. 4 shows the background density ($\hbox {f}_{{bg}}$) that is found to be 26.93 ± 0.25 stars $\hbox {arcmin}^{-2}$. The calculated values of the central density, the core radius and the border radius (R) are 21.06 ± 2.20 stars $\hbox {arcmin}^{-2}$, 1.05 ± 0.02 arcmin and 7.90 ± 0.21 arcmin, respectively, (see Table 1). The error of fitted parameters is calculated by using covariance matrix of curve_fit function in Scipy package (https://scipy.org/).

Another important parameter for the cluster is the star density contrast parameter, which is expressed as :-

3 $$\begin{aligned} \delta _c=1 \;+\; \dfrac{f_o}{f_{bg}} \end{aligned}$$

For King 18, the contrast parameter value is 1.75 which is smaller than the values ($7 \le \delta _c \le 23$) calculated for compact star clusters, as given by^40^. This means that King 18 is a sparse cluster.

We have estimated the cluster tidal radius according to the formula of^41^ :

4 $$\begin{aligned} R_t \;=\; 1.46 \times M_c^{1/3} \end{aligned}$$

where $R_t$ and $M_c$ are the tidal radius and total cluster mass, respectively. Our estimated value for the cluster tidal radius is 11.49 pc, where the total cluster mass is 487.39 $M_\odot$, see “The cluster mass”. This value is very high and we think the formula of^41^ is missing some masses compared to Gaia DR3 era (Gaia has higher limiting magnitude with wider G band).

There is another parameter in literature which is called limiting radius of cluster $r_{lim}$, that was introduced by^42^. The cluster limiting radius, $r_{lim}$, is calculated by comparing *f*(*r*) to a border background density level, $f_{b}$, defined as:

5 $$\begin{aligned} f_b = f_{bg} +3 \sigma _{bg} \end{aligned}$$

where $\sigma _{bg}$ is the uncertainty of $f_{bg}$. The $r_{lim}$ is calculated according to the following formula :

6 $$\begin{aligned} r_{lim} \;=\; r_c \; \sqrt{\frac{f_o}{3 \sigma _{bg}}-1} \end{aligned}$$

For our cluster King 18, this value is about 2.20 arcmin which is an unrealistic value. Moreover, the last equation (intrinsic value for cluster) depends on $\sigma _{bg}$ of the background and foreground stars which is non physical.

## Proper motion, membership determination and cluster center

The determination of essential parameters of a cluster is affected by the contamination due to field stars. The membership determination of stars in star clusters was carried out in previous years through photometric and kinematic data^43–45^. Recently, the astrometric data from Gaia survey has made the kinematic method of membership determination more trustworthy. Proper motion and parallax are very precious agents to separate field stars from the cluster zone, as cluster stars have similar kinematical properties and distances^46^. We have used Gaia DR3 proper motion and parallax data to separate cluster star members from non members.

**Table 1:** King model fit parameters.

Name	$\hbox {f}_o$	$\hbox {f}_{{bg}}$	$\hbox {r}_c$	$\delta _c$	radius
King 18	21.06 ± 2.20	26.93 ± 0.27	1.05 ± 0.02	1.78	7.90 ± 0.21

The Unsupervised Photometric Membership Assignment in Stellar Clusters algorithm (UPMASK), originally presented in^47^, has the advantage of being not only non-parametric, but also unsupervised.This means that no prior selection of field stars is necessary in order to serve as a comparison model. The *pyUPMASK* python package (https://github.com/msolpera/pyUPMASK)^48^ is an improved version of the original UPMASK algorithm. It relies on the Python library scikit-learn^49^ (https://scikit-learn.org/stable/) for the implementation of most of the supported clustering methods (https://github.com/adamdempsey90/StarClusters). This library includes around a dozen of different clustering methods for unlabeled data, which are all available to use in the *pyUPMASK*.

**Figure 5:**
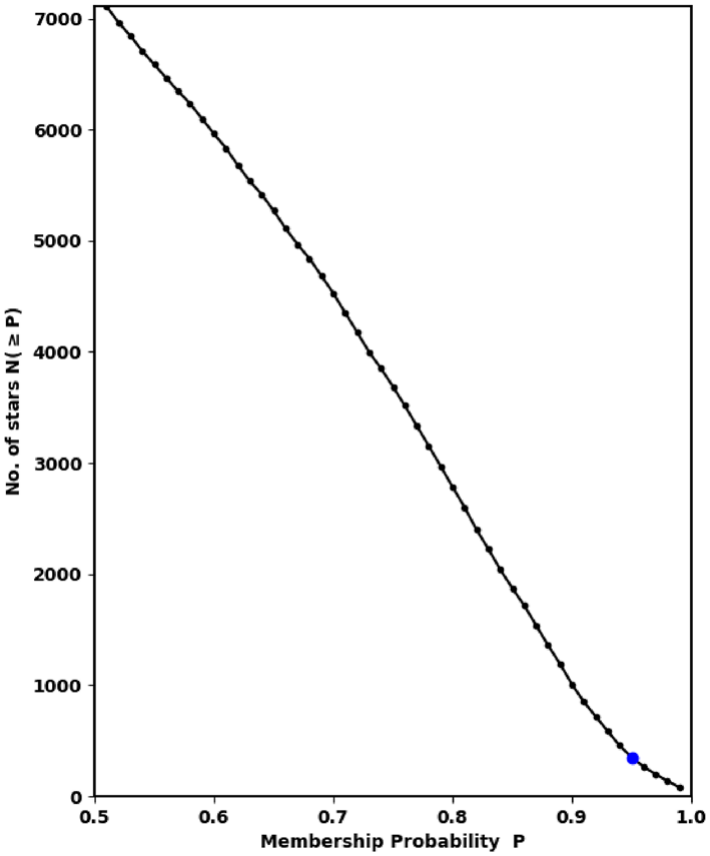
The number of stars as function of membership probability, the output of *pyUPMask* code.

We have used the *pyUPMASK* python package for finding the membership probability. We have fed it with Gaia DR3 data, about 21926 stars within $16^\prime$. Fig. 5 plots the total number of stars (N($\ge$P)) as a function of membership probability. From the fitting of King profile (“Cluster density profile and radius”), we get the total number of member stars as 307, which corresponds to a probability that is larger than 94%. This probability value is high because of contamination of the crowded field stars.

We have determined the cluster parameters by averaging the values of member stars with probability greater than 98% within 5 arcmin radius, to get more accuracy. The cluster center is found at $\alpha =343.044 \pm 0.048$; $22^{h}52^{m}10.48^{s}$ and $\delta =58.289 \pm 0.032$; $58^{d}17^{m}19.9^{s}$, which is corresponding to galactic l and b (${107.78}^{\circ }$, ${-1.03}^{\circ }$). In addition, the values of $\mu _{\alpha } cos\delta$ and $\mu _{\delta }$ are -2.603 ± 0.018 and -2.106 ± 0.013, respectively.

The average value of parallax ($\varpi$) is found to be 0.324 ± 0.040 mas. Thus the cluster distance ($d_{\varpi } (pc)\approx 1000./\varpi (mas)$) that corresponds to parallax, is 3.086 ± 0.038 kpc, which is in good agreement with our photometric data results within the errors, see Table 2 for tabulated results.

**Table 2:** The center’s coordinates of King 18.

Name	$\alpha$	$\delta$	$\mu _{\alpha }cos\delta$	$\mu _{\delta }$	${\varpi }$	l	b
Unit	Degrees	Degrees	mas $\hbox {year}^{-1}$	mas $\hbox {year}^{-1}$	mas	Degrees	Degrees
King 18	343.044 ± 0.048	58.289 ± 0.032	-2.603 ± 0.018	-2.106 ± 0.013	0.324 ± 0.040	${107.780}^{\circ }$	-${1.030}^{\circ }$

Other important parameter is the angle $\theta$ which is the angle or direction of cluster movement in $\mu _{\alpha } \cos _{\delta }$ and $\mu _{\delta }$ space and is given by, see Fig. 7:

7 $$\begin{aligned} \varvec{\theta }= \tan ^{-1}\left( \frac{\mu _{\delta }}{\mu _{\alpha } \cos \delta } \right) \end{aligned}$$

**Figure 6:**
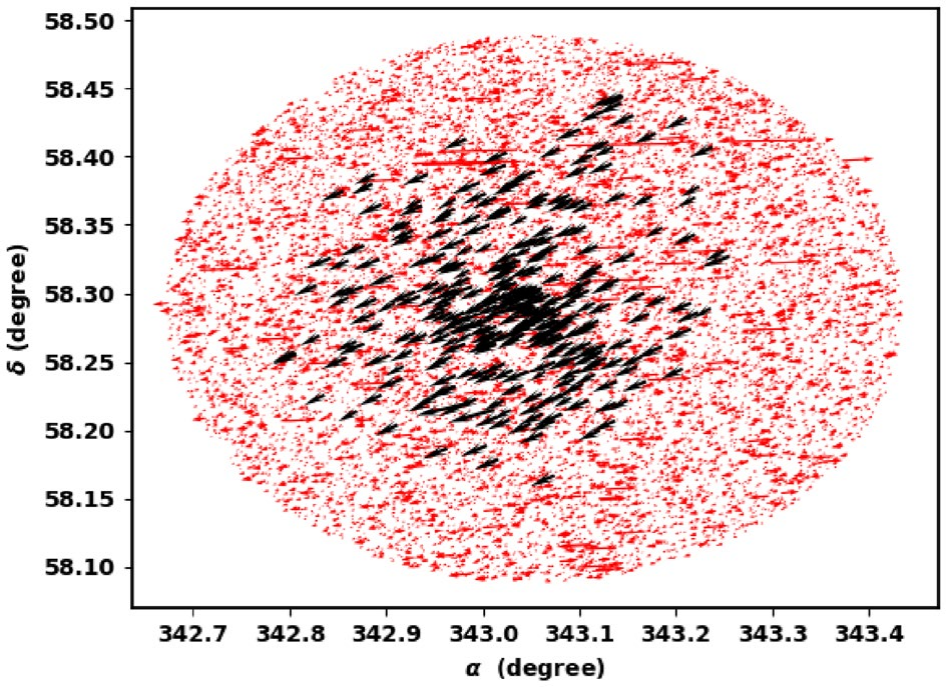
The co-moving stars of King 18 from Gaia DR3.

**Figure 7:**
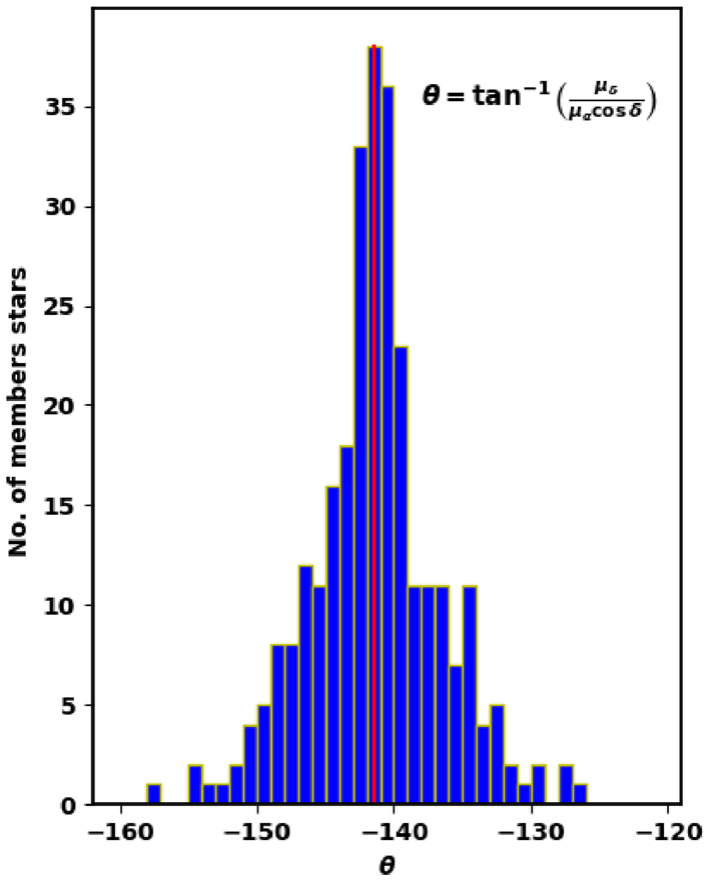
The $\theta$ histogram for member stars.

Cluster member stars will move nearly with the same direction through space. Figure 6 shows the $\theta$ histogram for member stars with average angle about $-140.9^{\circ } \pm 2.365^{\circ }$, which is more clear than Fig. 7. Moreover, dispersion in $\theta$ histogram depends on cluster age and how strongly the cluster is bound.

### Test of members’ separation method

In literature, the probability cut-off of member stars is often taken at 50% which is not correct. The probability cut-off value depends on the used method itself, as the density of the field and the distance between the star and the cluster’s center. Critically, the choice of the probability cut-off value must be carefully tested, otherwise we will get wrong member stars. On the other hand, the fitted King profile model can play important role in this context

**Figure 8:**
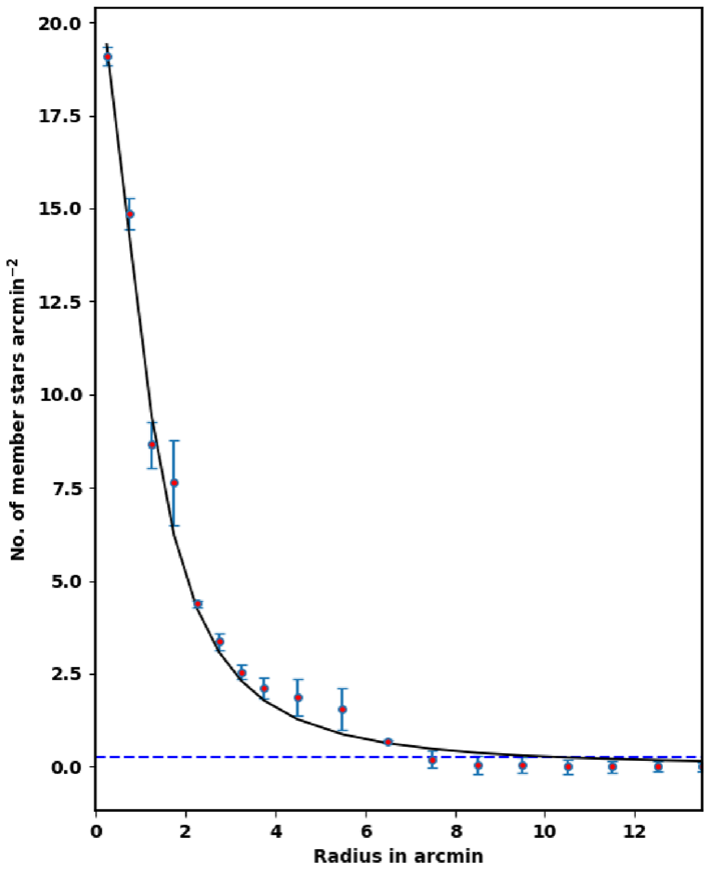
The stellar density profile of member stars. The solid line is the $f_o / (\; 1 + (r/rc)^2 \;)$ fitted King profile. The red dots are star density counts of member stars with probability of 94%.

**Figure 9:**
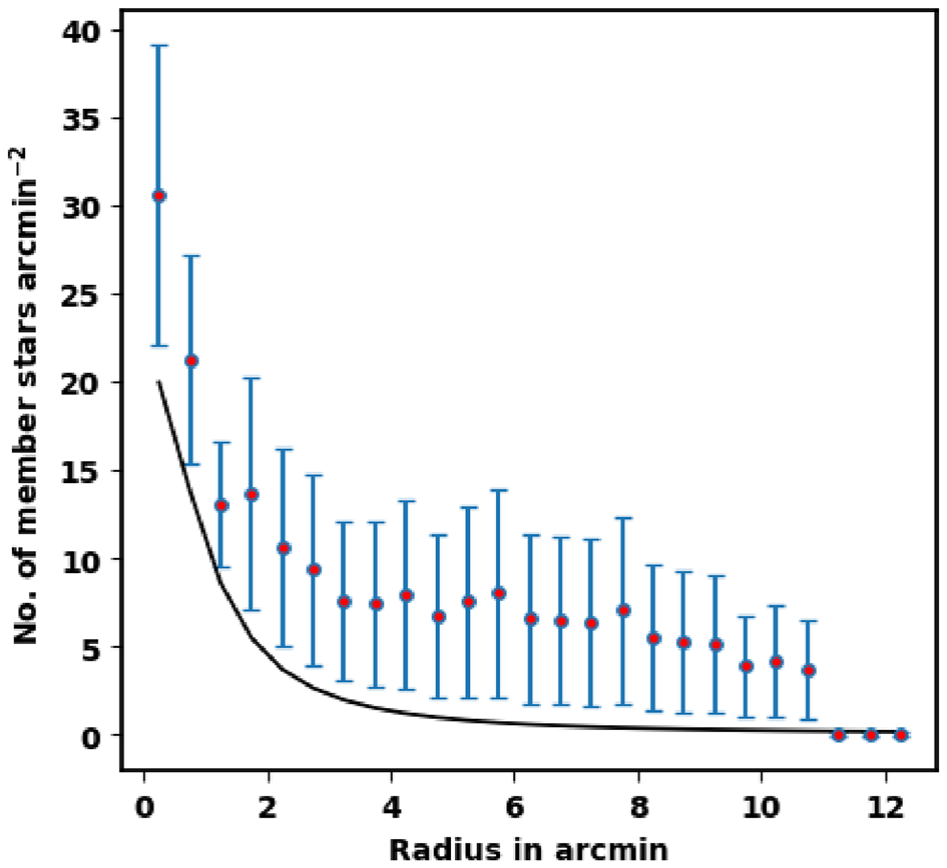
The cluster stellar density at probability cut-off value 80%. The members at this probability cut-off are much over King model function which are overestimated.

To test the probability cut-off value and the vitality of members’ separation method, we plot again the stellar density profile, but for member stars only as shown in Fig. 8. This result is very satisfactory and agrees with King profile. The King density profile can constrain both the vitality of membership separation method and the total number of member stars in the cluster. On the contrary, if the membership separation method or probability cut-off value are not correct, we will get member stars over or under estimation. As example, if the probability cut-off value is 80%, we will get overestimated member stars, as seen in Fig. 9. In future work, we will address this point in more detail.

## The color magnitude diagrams and cluster age

Color-magnitude diagrams (CMDs) of OCs introduce empirical isochrones to compare with the theoretical models of stellar evolution^50,51^. CMDs are efficient tools to estimate distance, age, and metallicity of an open cluster. Moreover, comparing the observed CMDs with the theoretical isochrones can also give wealth of information about masses of stars in an open cluster. The theoretical isochrones are downloaded from the CMD 3.7 web site (http://stev.oapd.inaf.it/cgi-bin/cmd) using PARSEC version 1.25 s^52^.

### Extinction

A precise interstellar dust extinction law is critically important to interpret observations. Extinction coefficients per passbands depend on the source spectral energy distribution, interstellar matter and on the extinction itself. Both the color excess ratio (CER) E($\lambda -\lambda _1$)/E($A_{\lambda _2}-A_{\lambda _1}$) and the relative extinction $A_\lambda /A_{\lambda _1}$ are indicators of the extinction law. Following the method presented in^53^, we compute the extinction coefficient in the Gaia and 2MASS bands by using the relation $A_{\lambda }= a A_V$, as an example $A_G/A_V=0.789$, $A_{BP}/A_V= 1.002$ and $A_{RP}/A_V=0.589$. For 2MASS, $A_J/A_V=0.243$, $A_{K_s}/A_V=.078$ and $A_{H}/A_V=0.131$.

For VRI observations, we use values of the extinction law of^54,55^, which are $A_U/A_V = 1.55814$, $A_B/A_V= 1.3262$, $A_R/A_V = 0.81$ and $A_I/A_v = 0.56$. Then we can get the relation between extinction and color excess as follow:

$$\begin{aligned} A_J&= 1.473 \times E(J-K_s) \\ A_G&= 1.84 \times E(G_{BP}-G_{RP}) \\ A_V&= 2.494 \times E(V-I) \\ A_V&= 3.1 \times E(B-V) \end{aligned}$$

From isochrone fitting we are able to estimate the color excess and finally extinction. By using the following equation the intrinsic distance modulus $(m-M)_o$ can be calculated :

8 $$\begin{aligned} \left( m-M \right) _{obs} = \left( m-M \right) _{o} + A_{\lambda } \end{aligned}$$

where *m* is the apparent absorbed magnitude, *M* is the absolute magnitude and $A_{\lambda }$ is the extinction in $\lambda$ band.

### The CMD Of Gaia DR3 data

**Figure 10:**
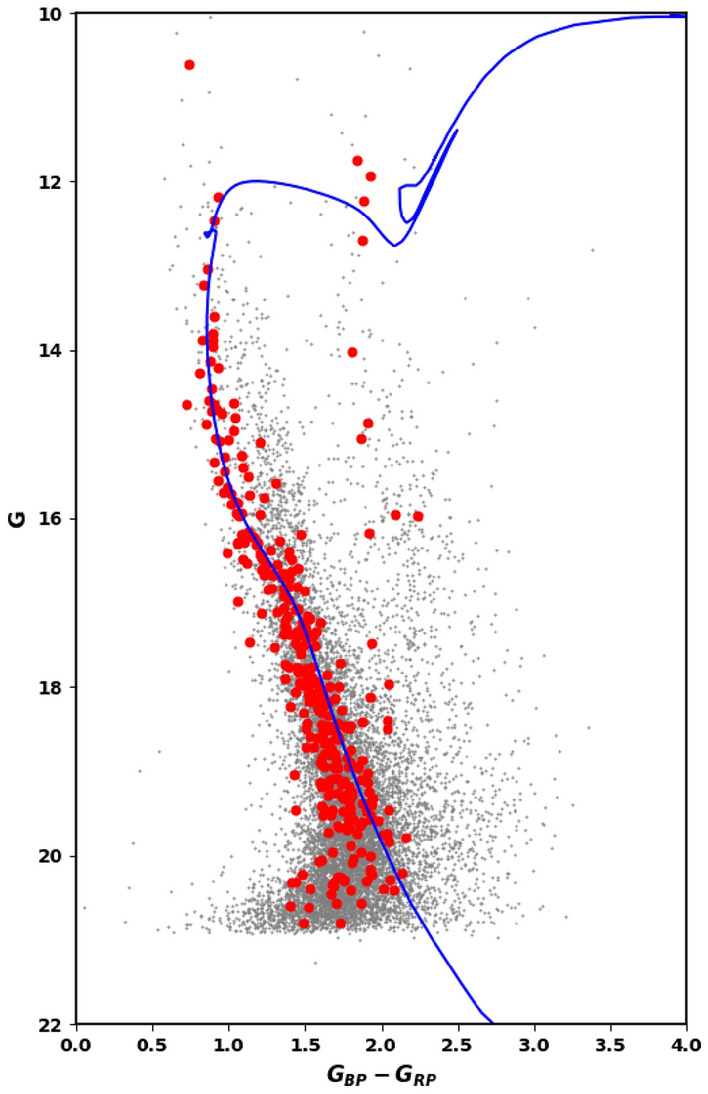
The color magnitude diagram (CMD) for the clusters’ members of King 18 using the photometric bands (G, $G_{BP}$ & $G_{RP}$) of the Gaia DR3.

By using the photometric data extracted from Gaia DR3 of stars of King 18, the CMD is plotted in Fig. 10. The CMD is fitted by the theoretical isochrones of^50^.

We have found that the observed distance modulus and the color excess E($\hbox {G}_{{BP}}$–$\hbox {G}_{{RP}}$) are 14.20 ± 0.80 mag and 0.98 ± 0.13 mag, respectively. To obtain the distance modulus $\left( m-M \right) _{o}$ and the extinction in G band $A_G$, we used the equation in “Extinction”. These values are found to be 12.38 and 1.82, respectively, which is corespondent to the distance $d_{iso}$ of 2992.0 ± 47 pc. Moreover the fitted isochrone produces a cluster age of about 224 ± 6.3 Myr, at Z = 0.0152. Figure 10 shows the CMD of King 18 using the wide photometric bands (G, $\hbox {G}_{{BP}}$ & $\hbox {G}_{{RP}}$) of the Gaia DR3 database.

### The CMD of 2MASS data

**Figure 11:**
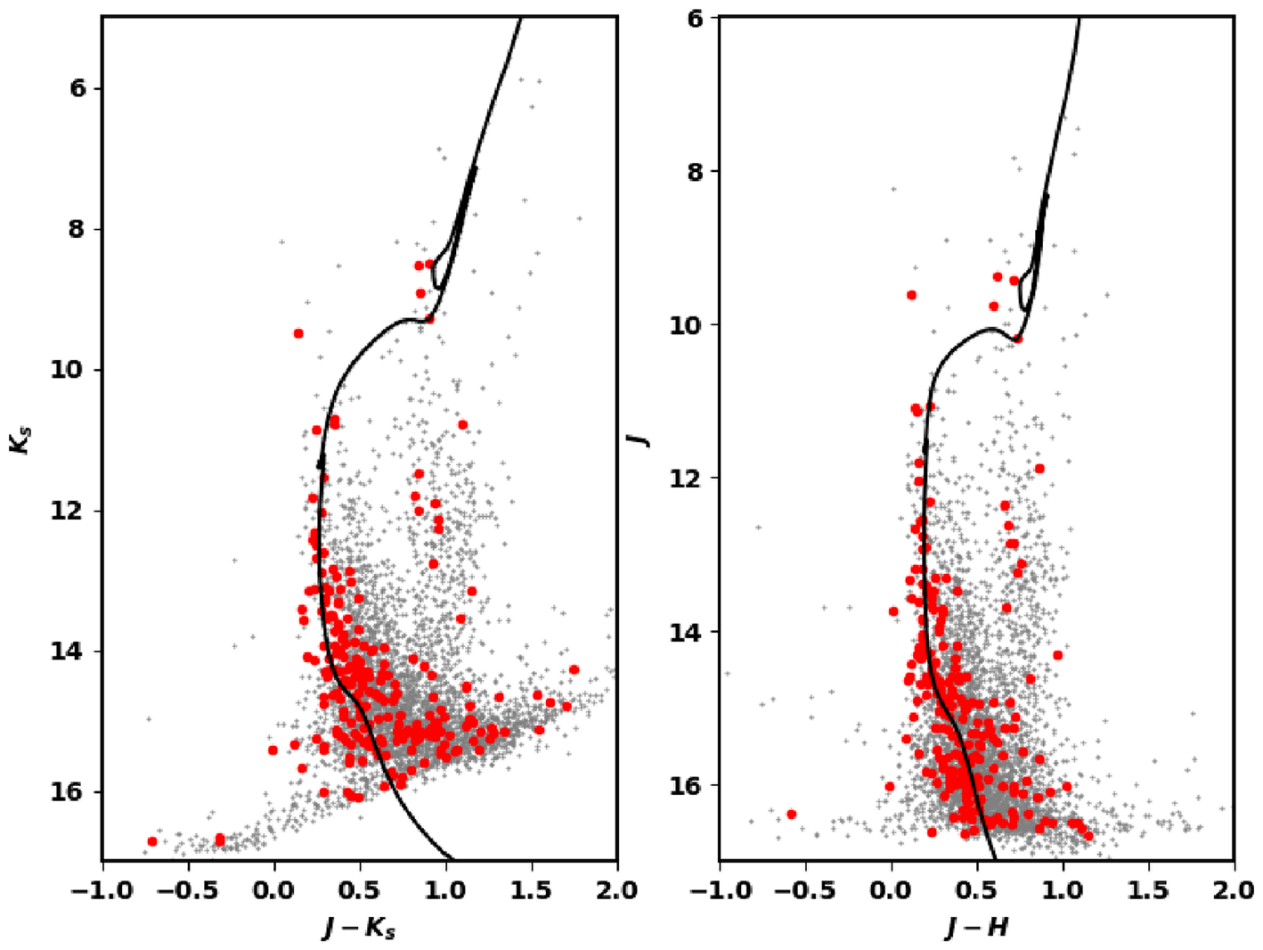
Left: the CMD $\hbox {K}_s$ and J-$\hbox {K}_s$ fit isochrone. Right: the J and J-H one.

Using the intersect1d function in the Python Numpy package (https://numpy.org/)^56^, we have matched member stars discovered in Gaia DR3 with 2MASS data. We found 231 member stars in both databases. The CMD of matched 2MASS data can check the vitality of membership separation method. Next, we plot the CMDs as shown in Fig. 11. From isochrone fitting, we obtain the color excess values of $E(J-K_s)$ and $E(J-H)$ as 0.380 ± 0.091 and 0.260 ± 0.036, respectively. Additionally, $(m-M)_{obs}$ in J band is 12.880 ± 0.202. According to equations in “Extinction ”, we get the $\hbox {A}_J$ value as 0.560 ± 0.081. At the end, the distance modulus is found to be 12.320 ± 0.107, which is corresponding to the distance value of 2910.00 ± 36.50 Pc. This value is in excellent agreement with Gaia parallax and photometry parameters.

### The CMD of VRI observation

**Figure 12:**
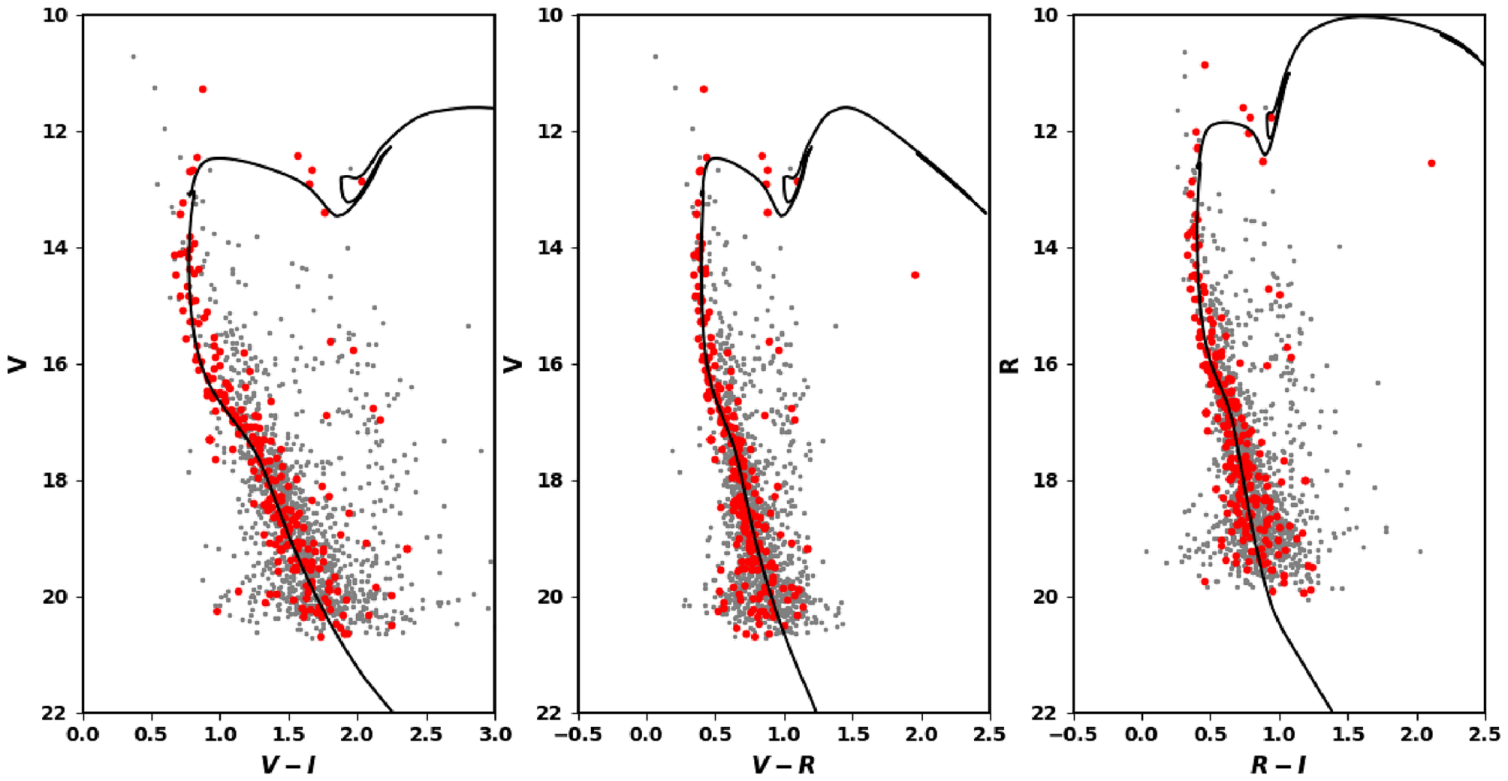
The color magnitude diagram (CMD) for the clusters’ members of King 18 using the photometric bands of VRI Observations.

Additionally, we cross-matched member stars identified by CCD VRI observations and Gaia. In both of them, we have found 278 stars, which is more than the outcome of cross-matching 2MASS and CCD VRI observations (231 stars). For isochrone fitting, we plot CMD V and (V–I) as in Fig. 12. We have found that the apparent distance modulus $(m-M)_{obs}$ and color excess E(V–I) are 14.4 $\pm .305$ and 0.85 $\pm .087$, respectively.

Using relationship in “Extinction”, the extinctions are found as $A_V=2.12$, $A_R=1.72$ and $A_I=1.27$. Next, we obtain the intrinsic distance modulus $(m-M)_o = 12.28 \pm 0.29$, that corresponds to the distance of 2857.5 pc. Moreover the age of used isochrones for fitting is $231\pm 23.4$ myr. At the end, the data reduction and analysis results of Gaia DR3, VRI observations and 2MASS are in agreement with each others within small errors.

## Luminosity, the cluster mass, mass functions and dynamical state

### Luminosity function

**Figure 13:**
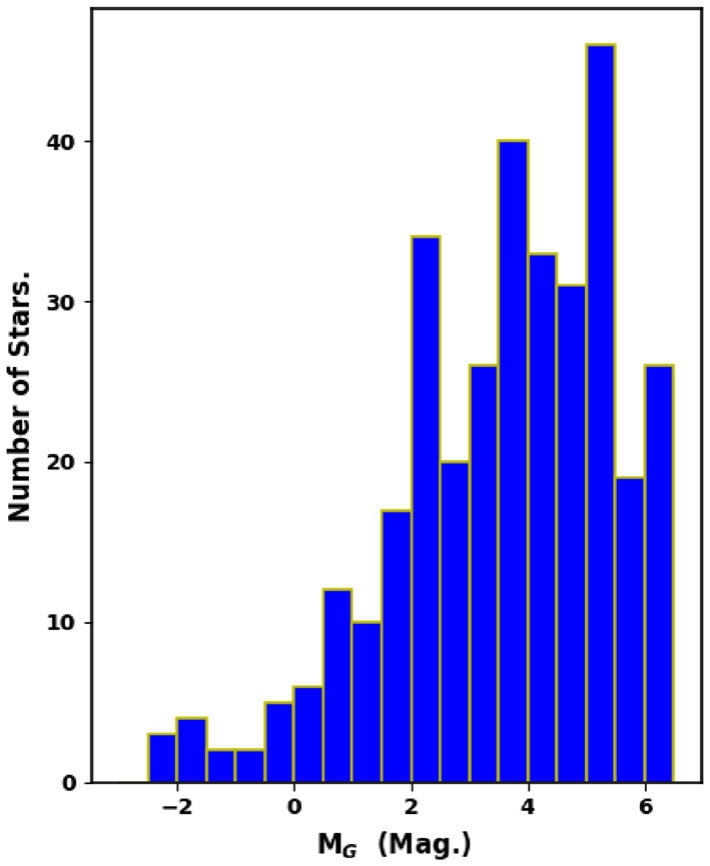
The luminosity function (LF) of King 18 with bin interval of 0.5 mag.

It is clear that luminosity and mass functions (LF & MF) are fundamentally dependent on the cluster’s membership. To remove field stars contamination completely from the main sequence stars of King 18, we used probable cluster members selected by using *pyUPMASK* python package. After that, we used the photometric data to obtain LF before estimating the MF. For the LF, we converted the apparent G magnitude of member stars into absolute magnitude. Then, we plot histograms (Fig. 13) showing the LF of King 18.

### The cluster mass

It is obvious that the individual star mass in a cluster is a very important parameter in addition to the total cluster mass as well. After making isochrone fitting, we get the absolute magnitude $\hbox {M}_G$ and the intrinsic color $G_{BP}-G_{RP}$. The mass obtained by the normal polynomial fitting is incorrect and yields misleading values. We need an interpolation routine with two independent variables. So we have used *SmoothBivariateSpline* routine in Python Scipy (https://scipy.org/) package^57^ which uses two variables for interpolation because the star mass depends on magnitude and color as well.

We have used $$M_G$$ and $$(G_{BP}-G_{RP})_o$$ as two independent interpolation variables from the best isochrone fit. By this way, we obtain the individual mass of every member star. Now, we are able to get the total cluster mass with high accuracy, $$M_c = $$ 487.39 $$M_\odot $$. Also we obtain the cluster mass profile as shown in Fig. 15. Moreover, we get $$R_h=2.73$$ pc, within which half of the cluster mass is included, see Eq. (10).

### Mass function

**Figure 14:**
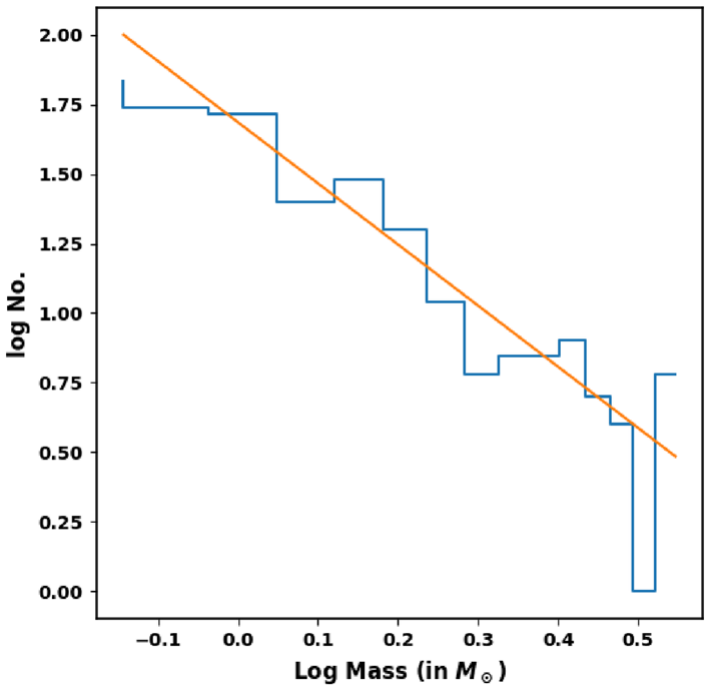
The initial mass function (IMF) of King 18.

The mass function (MF) can be defined as the distribution of masses of cluster’s stars per unit volume during the time of star formation. We can convert LF into MF by using a mass-luminosity relation. As we can not get an observational transformation, we must rely on theoretical models. In order to transform LF into MF, we use the theoretical isochrones of^50,58^. The topic concerning the initial mass function (IMF), whether it is universal in time and space or it depends on conditions of star formation, represents a current mystery^59–61^. Also, The study of mass-segregation in OCs provides an evidence for the distribution of low and high mass stars towards the cluster region. The IMF can be expressed as;

9 $$\begin{aligned} \dfrac{dN}{dM} \propto M^{-{\alpha }} \end{aligned}$$

where, $\dfrac{dN}{dM}$ is the number of stars that has a mass interval from M to M + dM, and $\alpha$ is the slope of the mass function. The value of $\alpha$ is found to be 2.27 ± 0.17 (see Fig. 14), which is in agreement with Salpeter value^62^.

### Dynamical state

**Figure 15:**
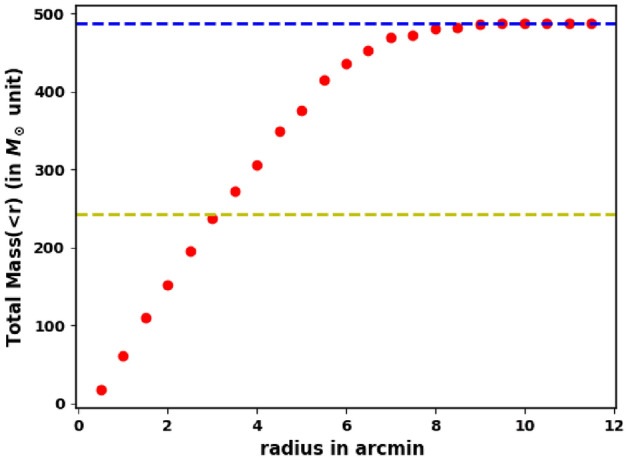
The mass profile M(<r) of King 18. The horizontal blue dashed line represents total mass and yellow dashed one represents the half cluster mass at $R_h$.

Another important parameter that is used to understand the dynamical evolution of a star cluster, is the relaxation time. It is the time scale in which the cluster will lose all its traces of initial conditions and its member stars will have a roughly Maxwellian distribution of velocities. According to^63^, the relaxation time is explained by,

10 $$\begin{aligned} T_R = \dfrac{8.9\times 10^5\sqrt{N}\times R_{h}^{1.5}}{\sqrt{m}\times \log (0.4N)} \end{aligned}$$

where N denotes for the number of cluster members, $\hbox {R}_h$ is the radius in pc, within which half of the cluster mass is included, and m is the average mass of the cluster in solar units. In Fig. 15, we plot the mass $M(>r)$ inside radius r. From this figure, we found the value of $R_h$ equals 3.11 arcmin (2.73 pc). By applying the above equation, we found that the relaxation time of King 18 equals 28.92 Myr which is much younger than the cluster age (224–251 Myr). That means that King 18 is dynamically stable and a relaxed cluster. Table 3 presents our results including all the calculated astrophysical parameters of Kink 18, in addition to a comparison with previous studies.

**Table 3:** Comparison between parameters of the open cluster King 18 in the current study and literature.

Parameter	Gaia DR3	Present work	^24^	^25^	^26^
		*VRI* photometric observations			
Radius (arcmin)	7.90 ± 0.21		9.50 ± 0.40 pc	4.80	8.00
Members	307		2400		
$\alpha$	${22}^h$ ${52}^m$ ${00}^s$		${22}^h$ ${52}^m$ ${03}^s$	${22}^h$ ${52}^m$ ${07}^s$	${22}^h$ ${52}^m$ ${01}^s$
$\delta$	+$\textrm{58}^\circ$ $\textrm{18}^\prime$ $\textrm{55}^\prime$ ${}^\prime$		+$\textrm{58}^\circ$ $\textrm{18}^\prime$00${}^\prime$ ${}^\prime$	+$\textrm{58}^\circ$ $\textrm{16}^\prime$ $\textrm{57}^\prime$ ${}^\prime$	+$\textrm{58}^\circ$ $\textrm{17}^\prime$36${}^\prime$ ${}^\prime$
$\mu _{\alpha }$cos$\delta \;\;$(mas/yr)	-2.603 ± 0.018				
$\mu$ ${}_{\delta }\;\;\;$ (mas/yr)	-2.106 ± 0.013				
Parallax $\varpi$ (mas)	0.324 ± 0.040				
E(B–V) (mag)			0.63 ± 0.01	0.52	0.69 ± 0.04
E(V–I) (mag)		0.850±0.087			
E(R–I) (mag)		0.45			
E($\hbox {G}_{{BP}}$–$\hbox {G}_{{RP}}$) (mag)	0.98 ± 0.13				
Age (Myr)	224 ± 6.3	224 ± 6.3	251.2	350	130 ± 10
Distance modulus (mag)	12.38±1.32	12.28 ±0.29	13.85	11.8	12.39 ± 0.21
Total mass ($\hbox {M}_\odot$)	487.39 $M_\odot$		1050		
MF slope $\alpha$	2.27±0.17		0.90 ± 0.50		
Relaxation time (Myr)	28.92				

## Summary and conclusions

We performed a study on the young open cluster King 18 based on Gaia DR3 photometric and astrometric data, and the *VRI* CCD photometric observations using the f/4.9 74-inch telescope at Kottamia Astronomical Observatory (KAO). According to our analysis for refining the fundamental parameters of King 18 in the Gaia era DR3 and VRI CCD observations, we presented a detailed astro-photometric study here, which is somehow different from the previous results. The main results of our analysis are as follow **:**

1. The slope value of King 18 mass function ($\alpha$) is found to be 2.27, which is in agreement with Salpeter value^62^. Based on our data of KAO and that of Gaia DR3, we have estimated the cluster age to be 224 ± 6.3 Myrs, and the relaxation time is 28.92 Myr. That means that King 18 is dynamically stable and relaxed cluster.
2. The cluster distance modulus from Gaia, 2Mass and VRI observations has been determined to be 12.38 ± 1.32, 12.32 ± 0.11 and 12.28 ± 0.29 mag respectively, which corresponds to distances of 2992.26, 2910.72 and 2857.59 pc, respectively. These results are in good agreement within the error. Moreover the color excess E(V–I), E(J–$\hbox {K}_s$) and E($\hbox {G}_{{BP}}$–$\hbox {G}_{{RP}}$) are 0.85 ± 0.09, 0.38 ± 0.09 and 0.98 ± 0.13, respectively.
3. The values of proper motion ($\mu _{\alpha }$cos$\delta$, $\mu _{\delta }$), and parallaxes ($\varpi$) are $-2.603 \pm 0.018$, $-2.106 \pm 0.013$ and $0.324 \pm 0.040$ respectively. The cluster distance corresponding to parallax ($\varpi$) is 3.236 ± 0.500 kpc which is in good agreements with our photometric data result within the errors.

Finally, the present and previous results are summarized and compared with others in Table 3.

## Data Availability

Our VRI CCD observations: are available upon request from any of the authors (nasser_ahnmed@yahoo.com) Gaia and 2Mass data: are available for free in webpage https://vizier.cds.unistra.fr/ .
